# Clinical and immunological correlates of elevated age-adjusted QAlb in anti-NMDAR encephalitis

**DOI:** 10.3389/fnhum.2026.1914297

**Published:** 2026-08-07

**Authors:** Binting Liu, Yuqian Zhang, Yifang Ma, Xiaojiao Ci, Jie Lu

**Affiliations:** Department of Neurology, The Affiliated Brain Hospital of Nanjing Medical University, Nanjing, China

**Keywords:** anti-N-methyl-D-aspartate receptor encephalitis, blood-CSF barrier, cerebrospinal fluid/serum albumin quotient, intrathecal IgG synthesis, reibergram

## Abstract

**Background:**

Anti-NMDAR encephalitis is a common form of autoimmune encephalitis with marked clinical heterogeneity. The cerebrospinal fluid (CSF)/serum albumin quotient (QAlb) is a clinically accessible marker reflecting blood-CSF barrier (BCB) function. However, the clinical significance of elevated age-adjusted QAlb in anti-NMDAR encephalitis remains unclear.

**Methods:**

We retrospectively enrolled 103 patients with anti-NMDAR encephalitis and classified them into Elevated QAlb and Normal QAlb groups according to whether measured QAlb exceeded its age-adjusted upper reference limit (Qlim). Clinical features, paired serum-CSF findings, quantitative indices of intrathecal IgG synthesis, MRI/EEG findings, disease severity, and discharge outcomes were compared. Logistic regression, sensitivity analyses, Reibergram, and Spearman correlation analyses were performed.

**Results:**

Elevated age-adjusted QAlb was identified in 36 patients (35.0%). Compared with the Normal QAlb group, the Elevated QAlb group had higher proportions of male patients and prodromal headache, and showed higher CSF protein, CSF albumin, CSF IgG, IgG index (IgGI), and 24-h intrathecal IgG synthesis rate. Reibergram analysis showed that a higher proportion of patients in the Elevated QAlb group were above the Qlim(IgG) curve than in the Normal QAlb group [9/36 (25.0%) vs. 4/67 (6.0%), *p* = 0.010], supporting increased total intrathecal IgG synthesis beyond passive blood-derived transfer in a subset of patients. In contrast, CSF anti-NMDAR antibody titers, CSF pleocytosis, peak mRS and CASE scores, ICU admission, and discharge outcomes were comparable between groups. Male sex and prodromal headache remained associated with elevated QAlb after multivariable adjustment. Hippocampal MRI abnormalities were more frequent in the Elevated QAlb group, but this finding was based on only nine affected patients and should be considered exploratory.

**Conclusion:**

Elevated age-adjusted QAlb was associated with increased quantitative markers of total intrathecal IgG synthesis, but not with CSF anti-NMDAR antibody titers, acute disease severity, or short-term discharge outcomes. These findings support interpreting QAlb as an accessible CSF marker related to BCB function rather than as a standalone acute prognostic marker. The observed association with hippocampal MRI abnormalities was exploratory and requires independent validation.

## Introduction

Anti-N-methyl-D-aspartate receptor (NMDAR) encephalitis is one of the most common types of autoimmune encephalitis, primarily mediated by pathogenic autoantibodies against the GluN1 subunit of the NMDAR (Dalmau et al., 2007). Since its first systematic description in 2007, clinical recognition and research focus on this disease have expanded substantially (Dalmau et al., 2007; Dalmau et al., 2011). The clinical presentation typically manifests as a complex and multiphasic neuropsychiatric syndrome that includes psychiatric symptoms, seizures, cognitive impairment, movement disorders, decreased level of consciousness and autonomic dysfunction (Dalmau et al., 2019). While autoantibody-induced impairment of synaptic NMDAR signaling constitutes the core pathogenic mechanism of anti-NMDAR encephalitis, patients exhibit marked interindividual differences in disease progression, acute severity, therapeutic response and long-term recovery (Cai et al., 2021; Gresa-Arribas et al., 2014). Furthermore, CSF anti-NMDAR antibody titers fail to fully account for such clinical heterogeneity, implying that additional immunological mediators and central nervous system (CNS) microenvironmental alterations jointly shape disease manifestations.

Among the potential pathophysiological mechanisms underlying anti-NMDAR encephalitis, the integrity of blood–CNS interfaces emerges as an important factor in disease initiation and progression (Gong et al., 2023). These interfaces include the blood–brain barrier (BBB), formed mainly by tight junctions between brain microvascular endothelial cells, and the BCB, formed by tight junctions between choroid plexus epithelial cells (Hladky and Barrand, 2016). The former primarily regulates molecular exchange between blood and brain interstitial fluid, while the latter is responsible for active secretion of CSF and exchange between blood and CSF (Redzic, 2011). In anti-NMDAR encephalitis, emerging evidence suggests that barrier-related endothelial or choroid plexus epithelial dysfunction may participate in disease-related neuroinflammation (Gong et al., 2023; Shu et al., 2023). Therefore, evaluating the integrity of the CNS barriers provides an alternative perspective for understanding anti-NMDAR encephalitis.

While advanced neuroimaging and tracer-based techniques can evaluate barrier permeability (Heye et al., 2014; Sweeney et al., 2019), they are often restricted in routine clinical practice by cost, protocol heterogeneity and retrospective data completeness. Alternatively, the QAlb is widely used as an accessible CSF marker derived from paired serum and CSF samples. Because albumin is synthesized outside the CNS, an increased QAlb primarily reflects increased blood-to-CSF albumin transfer and altered BCB function (Deisenhammer et al., 2006). In addition, QAlb increases physiologically with age, probably reflecting age-related changes in barrier function and CSF turnover (Brettschneider et al., 2005). For this reason, the Qlim provides an individualized benchmark for determining whether the measured QAlb exceeds the expected range for a given age. This approach is particularly critical in relatively young patient cohorts, in which conventional fixed thresholds may underestimate mild QAlb abnormalities because of a lower physiological baseline. Elevated QAlb has been investigated in several immune-mediated neurological diseases, including multiple sclerosis (MS), neuromyelitis optica spectrum disorder (NMOSD) and myelin oligodendrocyte glycoprotein antibody-associated disease (MOGAD) (Uher et al., 2016; Zhang et al., 2025; Johnsson et al., 2025). Across these conditions, QAlb abnormalities have been variably associated with inflammatory activity, disease severity, relapse and clinical outcome. In anti-NMDAR encephalitis, however, studies specifically addressing QAlb remain limited, and their findings regarding disease severity and short-term outcome have been inconsistent (Yu et al., 2021; Qiao et al., 2024; Titulaer et al., 2013). These discrepancies may be partly attributable to differences in cohort composition, disease severity distribution, timing of lumbar puncture, treatment timing, outcome assessment and the use of fixed rather than age-adjusted QAlb thresholds. In addition, the relationship between age-adjusted QAlb and quantitative indices of intrathecal IgG synthesis has not been fully characterized. Therefore, we stratified patients according to whether measured QAlb exceeded the Qlim. We systematically compared demographic characteristics, clinical manifestations, CSF immunological markers, neuroimaging and EEG findings, and short-term outcomes between groups. Reibergram and correlation analyses were additionally used to characterize the relationship between QAlb and intrathecal IgG synthesis beyond passive blood-derived transfer. This study aimed to clarify the clinical associations of elevated age-adjusted QAlb and to explore its relationship with intrathecal immune activation and disease characteristics in anti-NMDAR encephalitis.

## Materials and methods

### Patients

**Figure 1:**
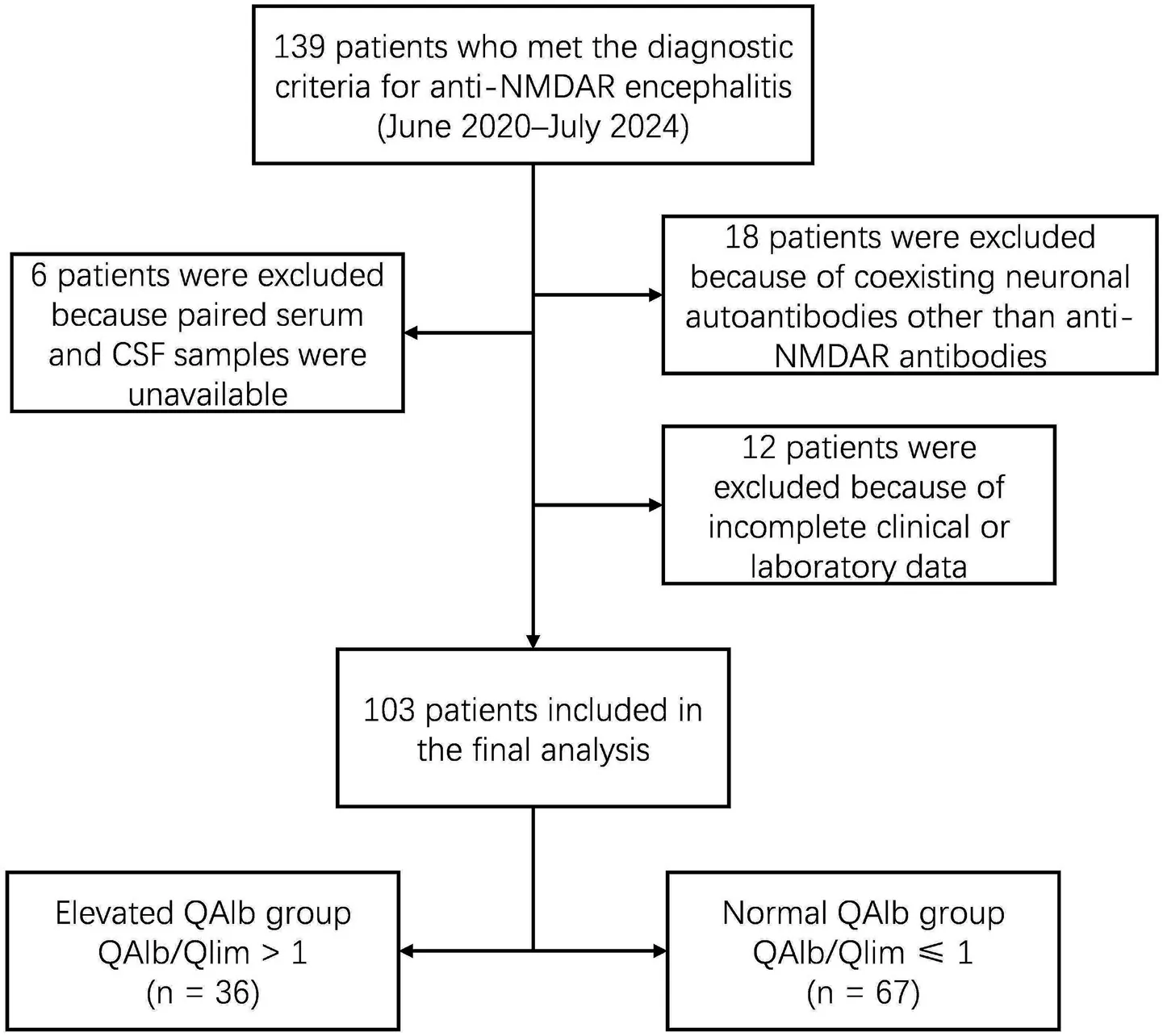
Study flow diagram. A total of 139 consecutive patients fulfilling the diagnostic criteria for anti-NMDAR encephalitis between June 2020 and July 2024 were assessed for eligibility. After exclusion of patients without paired pre-immunotherapy serum and CSF samples, patients with coexisting neuronal antibodies other than anti-NMDAR antibodies, and patients with incomplete clinical or laboratory data, 103 patients were included in the final analysis. Among them, 36 were classified into the Elevated QAlb group and 67 into the Normal QAlb group according to the QAlb/Qlim ratio.

This was a retrospective study involving patients with anti-NMDAR encephalitis from June 2020 to July 2024 in the Affiliated Brain Hospital of Nanjing Medical University. All consecutive hospitalized patients fulfilling the diagnostic criteria for anti-NMDAR encephalitis during the study period were assessed for eligibility. The inclusion criteria were as follows: (1) fulfillment of the 2016 diagnostic criteria for anti-NMDAR encephalitis proposed by Graus et al. (2016), with anti-NMDAR antibodies detected by cell-based assay (CBA) in CSF; (2) paired serum and CSF samples obtained on the same day prior to immunotherapy. The exclusion criteria were: (1) CNS infection due to viral, bacterial or other causes; (2) coexisting antibody-positive autoimmune encephalitis with neuronal antibodies other than anti-NMDAR antibodies, or systemic autoimmune diseases; (3) other severe neurological or primary psychiatric disorders that could substantially confound clinical assessment, such as brain tumor, stroke, myasthenia gravis, or schizophrenia. (4) incomplete clinical or laboratory data. All included patients underwent lumbar puncture before immunotherapy (Figure 1).

### Clinical data collection

Demographic and clinical data were retrospectively extracted from electronic medical records. The collected variables included sex, age at disease onset, history of viral encephalitis (VE), ovarian teratoma, serum anti-NMDAR antibody status, prodromal symptoms, initial symptoms, core clinical manifestations during hospitalization, treatment strategies, and short-term functional outcomes. Prodromal symptoms included fever and headache. Initial symptoms included psychiatric symptoms, seizures, and speech dysfunction. Core clinical manifestations during hospitalization included psychiatric symptoms, seizures, cognitive impairment, speech dysfunction, movement disorders, autonomic dysfunction, decreased level of consciousness, and central hypoventilation. The time intervals from symptom onset to hospital admission, lumbar puncture, and initiation of immunotherapy were recorded.

MRI and EEG findings were also incorporated into the analysis, independently evaluated by senior neurologists and radiologists who were blinded to QAlb group status. Brain MRI was performed using 3.0-T scanners within 3 days after admission as part of the routine diagnostic evaluation. MRI sequences generally included T1-weighted, T2-weighted, FLAIR, and diffusion-weighted sequences; contrast-enhanced images were evaluated when available. MRI abnormalities were categorized by anatomical location, including frontal lobe, parietal lobe, temporal lobe, hippocampal region, and cerebellar involvement. EEG findings were categorized as diffuse or focal slow waves, epileptiform discharges, or extreme delta brush patterns.

First-line immunotherapy included corticosteroids, intravenous immunoglobulin (IVIG), plasma exchange or their combinations. Second-line immunotherapy included rituximab, cyclophosphamide or other immunosuppressive treatments. Admission to intensive care unit (ICU) was also recorded as an indicator of disease severity.

Functional status was assessed using the modified Rankin Scale (mRS). Peak mRS represented the greatest functional disability during hospitalization, whereas discharge mRS was used to assess functional status at discharge. A discharge mRS score of 0–2 was defined as a favorable outcome, and a score of 3–6 was defined as a poor discharge outcome (van Swieten et al., 1988). Disease severity was additionally assessed using the Clinical Assessment Scale in Autoimmune Encephalitis (CASE), a disease-specific scale covering the major clinical domains of autoimmune encephalitis (Lim et al., 2019). Peak CASE score represented maximal acute disease severity, whereas discharge CASE score was used as an additional measure of clinical status at discharge. Peak mRS and peak CASE scores were retrospectively assigned by two trained neurologists based on detailed review of the medical records. Any initial discrepancies in scoring between the two evaluators were resolved through a joint re-examination of the medical records and detailed case discussions until a final consensus was reached.

### Serum and CSF analysis

Paired serum and CSF samples were obtained on the same day before immunotherapy. Anti-NMDAR antibodies were measured by Euroimmun CBA. CSF anti-NMDAR antibody positivity was defined as a titer ≥1:1, whereas serum anti-NMDAR antibody positivity was defined as a titer ≥1:10. Each antibody titer was first converted to its corresponding denominator value, and the natural logarithm of the denominator was then calculated. Routine CSF parameters including white blood cell count, total protein, glucose and chloride were obtained. CSF pleocytosis was defined as a white blood cell count >5 cells/μL. Albumin and IgG concentrations in paired serum and CSF samples were measured using standard turbidimetric assays.

QAlb was calculated as the ratio of CSF albumin to serum albumin (Deisenhammer et al., 2006). Because albumin is synthesized exclusively outside the CNS, QAlb provides a clinical measure of blood-to-CSF albumin transfer and BCB function (Deisenhammer et al., 2006; Reiber, 1994). Compared with total CSF protein, QAlb can correct for baseline serum albumin levels and exhibits higher specificity in evaluating the trans-barrier transport of blood-derived proteins. QAlb displays prominent age dependence and rises progressively during physiological aging, which is largely attributed to alterations in barrier function and CSF turnover rate (Castellazzi et al., 2020). To reduce the potential influence of age-related physiological variation, the Qlim was calculated using the Reiber formula: (4 + age/15) × 10^–3^ (Reiber, 1994). The QAlb/Qlim ratio was then calculated to express each patient’s measured QAlb relative to the expected upper reference limit for that age. A QAlb/Qlim ratio> 1 indicated that the measured QAlb exceeded the age-adjusted physiological upper reference limit (Zhang et al., 2025; Lai et al., 2022). Participants were thereby divided into the Elevated QAlb group and the Normal QAlb group.

Intrathecal IgG synthesis was comprehensively evaluated using the following quantitative indices: (1) IgG index (IgGI), calculated as (CSF IgG × serum albumin)/(serum IgG × CSF albumin) (Deisenhammer et al., 2006). An IgGI greater than 0.70 was considered elevated (Tibbling et al., 1977). (2) 24-h intrathecal IgG synthesis rate, determined by the formula: [(CSF IgG − serum IgG/369) − (CSF albumin − serum albumin/230) × (serum IgG / serum albumin) × 0.43] × 4. Values above 3.3 mg/24 h were considered elevated (Tourtellotte et al., 1985). Negative calculated values may occur when the measured CSF IgG concentration is lower than the amount predicted from passive transfer, and should be interpreted as absence of detectable excess IgG synthesis rather than as negative IgG production. QIgG was calculated as the ratio of CSF IgG to serum IgG. Reibergram analysis was performed to visualize the relationship between QAlb and QIgG and to further assess intrathecal IgG synthesis (Reiber and Lange, 1991). In the Reibergram, Qlim(IgG) represents the upper reference limit of QIgG expected from passive blood-derived IgG transfer at a given QAlb level and was calculated according to the Reiber hyperbolic function: Qlim(IgG) = 0.93 × √(QAlb^2^ + 6 × 10^−6^) − 1.7 × 10^–3^ (Reiber et al., 2004). For each patient, QIgG was compared with the corresponding Qlim(IgG) value. Patients with QIgG values above the Qlim(IgG) curve were considered to have intrathecal IgG synthesis exceeding that expected from passive blood-derived IgG transfer.

In addition, peripheral blood samples were obtained within 24 h of admission to measure hematological and inflammatory indices, including neutrophil, lymphocyte, and monocyte counts, as well as their derivatives: neutrophil-to-lymphocyte ratio (NLR), monocyte-to-lymphocyte ratio (MLR), and platelet-to-lymphocyte ratio (PLR).

### Statistical analysis

Statistical analyses were performed using SPSS (version 27.0) and R software (version 4.4.1). Data following a normal distribution are expressed as mean ± standard deviation. Data not following a normal distribution are expressed as median and interquartile range (IQR). Between-group comparisons for continuous variables were performed using Student’s *t*-test for normally distributed data or Mann–Whitney U test for non-normally distributed data. Categorical variables were analyzed using chi-square tests or Fisher’s exact tests as appropriate. Two-sided nominal *p* values <0.05 were considered statistically significant.

Univariable logistic regression was used to identify factors associated with elevated QAlb. Candidate variables included demographic characteristics, antibody-related variables, timing variables, prodromal symptoms, initial symptoms, core clinical manifestations, peripheral blood parameters, serum electrolytes, MRI and EEG findings, and acute disease-severity measures. Variables directly involved in the calculation or interpretation of QAlb, including CSF albumin, CSF protein, QAlb/Qlim, and calculated IgG synthesis indices, were excluded to avoid circularity or mathematical coupling. Variables with nominal *p* < 0.05 in univariable analyses were entered into an exploratory multivariable logistic regression model. To reduce overfitting, the number of covariates was restricted. Sensitivity analyses were performed by separately adding peak mRS, peak CASE score, time from symptom onset to hospital admission, time from symptom onset to lumbar puncture, or time from symptom onset to immunotherapy to the main model.

Given the exploratory nature of this study and the large number of comparisons, Benjamini–Hochberg false discovery rate (FDR) correction was additionally applied. Between-group comparisons in Tables 1–4 and full univariable logistic regression analyses were adjusted separately within their respective analysis families. Nominal *p* values were reported as the primary results, whereas FDR-adjusted *p* values were used to assess the robustness of exploratory findings.

**Table 1:** Baseline characteristics of patients with anti-NMDAR encephalitis in the Elevated QAlb and Normal QAlb groups.

Variable	Total (*n* = 103)	Elevated QAlb group (*n* = 36)	Normal QAlb group (*n* = 67)	*p* value	FDR-adjusted *p* value
Age at onset, years	26.0 (19.0,34.0)	31.5 (21.3,39.3)	24.0 (18.0,31.0)	0.020	0.109
Sex					
Male	40 (38.8%)	21 (58.3%)	19 (28.4%)	0.003	0.030
Female	63 (61.2%)	15 (41.7%)	48 (71.6%)		
Ovarian teratoma	13 (12.6%)	2 (5.6%)	11 (16.4%)	0.203	0.580
History of VE	11 (10.7%)	4 (11.1%)	7 (10.4%)	1.000	1.000
Positive for serum antibody	72 (69.9%)	26 (72.2%)	46 (68.7%)	0.707	0.974
Time from onset to hospitalization, days	10.0 (4.0,20.3)	12.0 (3.0,21.2)	10.0 (4.0,20.8)	0.975	1.000
Time from onset to lumbar puncture, days	15.0 (9.0,24.0)	17.0 (9.0,31.0)	14.0 (9.0,23.5)	0.284	0.710
Time from onset to immunotherapy, days	21.0 (12.0,29.5)	24.0 (14.0,35.0)	19.5 (12.0,26.3)	0.161	0.502
ICU admission	36 (35.0%)	9 (25.0%)	27 (40.3%)	0.121	0.427

Data are presented as median (interquartile range) or *n* (%), as appropriate. Time-related variables were calculated from symptom onset. QAlb, CSF/serum albumin quotient; VE, viral encephalitis; ICU, intensive care unit.

**Table 2:** Clinical characteristics of patients with anti-NMDAR encephalitis in the elevated QAlb and normal QAlb groups.

Variable	Total (*n* = 103)	Elevated QAlb group (*n* = 36)	Normal QAlb group (*n* = 67)	*p* value	FDR-adjusted *p* value
Prodromal symptom					
Headache	26 (25.2%)	15 (41.7%)	11 (16.4%)	0.005	0.037
Fever	30 (29.1%)	12 (33.3%)	18 (26.9%)	0.491	0.866
Initial symptom					
Psychiatric symptom	68 (66.0%)	24 (66.7%)	44 (65.7%)	0.919	1.000
Seizures	26 (25.2%)	9 (25.0%)	17 (25.4%)	0.967	1.000
Speech dysfunction	8 (7.80%)	3 (8.3%)	5 (7.5%)	1.000	1.000
Clinical presentation					
Psychiatric symptom	95 (92.2%)	33 (91.7%)	62 (92.5%)	1.000	1.000
Seizures	71 (68.9%)	23 (63.9%)	48 (71.6%)	0.418	0.782
Cognitive impairment	76 (73.8%)	24 (66.7%)	52 (77.6%)	0.228	0.622
Speech dysfunction	52 (50.5%)	17 (47.2%)	35 (52.2%)	0.627	0.965
Decreased level of consciousness	38 (36.9%)	10 (27.8%)	28 (41.8%)	0.160	0.508
Movement disorder	57 (55.3%)	18 (50.0%)	39 (58.2%)	0.424	0.782
Autonomic dysfunction	69 (67.0%)	21 (58.3%)	48 (71.6%)	0.171	0.513
Central hypoventilation	19 (18.4%)	6 (16.7%)	13 (19.4%)	0.733	0.774

Data are presented as *n* (%). QAlb, CSF/serum albumin quotient.

**Table 3:** CSF, serum, peripheral blood, MRI and EEG findings in the elevated QAlb and normal QAlb groups.

Variable	Total (*n* = 103)	Elevated QAlb group (*n* = 36)	Normal QAlb group (*n* = 67)	*p* value	FDR-adjusted *p* value
CSF antibody titer	1:10 (1:3.2,1:32)	1:10 (1:3.2,1:32)	1:10 (1:3.2,1:32)	0.760	0.974
CSF protein, g/L	0.43 (0.32,0.59)	0.67 (0.53,0.96)	0.37 (0.30,0.44)	<0.001	<0.001
CSF glucose, mmol/L	3.4 (3.1,3.8)	3.2 (2.9,3.8)	3.4 (3.1,3.7)	0.430	0.782
CSF chloride, mmol/L	121.4 (119.8,123.9)	121.2 (118.7,123.8)	121.4 (120.0,124.3)	0.343	0.762
CSF pleocytosis	59 (57.3%)	22 (61.1%)	37 (55.2%)	0.565	0.914
CSF albumin, mg/L	193.0 (140.0,277.0)	337.0 (256.0,504.8)	159.0 (129.0,193.0)	<0.001	<0.001
CSF IgG, mg/L	32.1 (22.7,53)	64.5 (47.3,98.2)	25.1 (20.3,32.1)	<0.001	<0.001
Serum albumin, g/L	41.09 ± 4.17	39.98 ± 4.33	41.68 ± 3.99	0.048	0.206
Serum lgG, g/L	10.89 (9.21,11.86)	10.00 (9.00,11.69)	10.97 (9.34,12.43)	0.309	0.742
IgGI	0.62 (0.51,0.77)	0.70 (0.57,1.00)	0.59 (0.50,0.73)	0.005	0.037
IgGI elevated	37 (35.9%)	18 (50.0%)	19 (28.4%)	0.029	0.145
24hIgG, mg/24 h	0.31 (−2.63,5.71)	6.09 (2.04,20.00)	−1.63 (−3.20,1.07)	<0.001	<0.001
24hIgG elevated	36 (35.0%)	26 (72.2%)	10 (14.9%)	<0.001	<0.001
WBC, 10^9/L	9.26 (7.30,11.86)	8.91 (7.48,11.87)	9.45 (6.93,11.86)	0.909	1.000
Neutrophil, 10^9/L	7.12 (5.12,9.17)	6.69 (5.25,9.13)	7.26 (4.93,9.32)	0.782	0.974
Monocyte, 10^9/L	0.58 (0.41,0.70)	0.58 (0.42,0.67)	0.58 (0.39,0.74)	0.793	0.974
Lymphocyte, 10^9/L	1.55 (1.24,1.96)	1.57 (1.25,1.89)	1.52 (1.14,2.06)	0.763	0.974
Platelet, 10^9/L	237 (195,282)	208 (171,256)	247 (215,292)	0.012	0.080
MLR	0.37 (0.24,0.50)	0.38 (0.24,0.49)	0.36 (0.25,0.53)	0.812	0.974
NLR	4.39 (2.93,6.96)	4.10 (2.86,7.06)	4.72 (3.08,6.96)	0.332	0.762
PLR	146.39 (119.74,197.00)	135.77 (102.49,171.41)	156.12 (121.23,225.77)	0.036	0.166
Na^+^, mmol/L	140.40 (138.20,143.00)	140.25 (137.75,142.50)	140.80 (138.20,143.40)	0.382	0.782
K^+^, mmol/L	3.77 (3.51,4.04)	3.74 (3.49,4.01)	3.77 (3.52,4.05)	0.719	0.974
Ca^2+^, mmol/L	2.29 (2.20,2.37)	2.27 (2.21,2.35)	2.30 (2.20,2.38)	0.399	0.782
Abnormal MRI					
Frontal lobe	14 (13.6%)	6 (16.7%)	8 (11.9%)	0.714	0.974
Parietal lobe	7 (6.8%)	4 (11.1%)	3 (4.5%)	0.387	0.782
Temporal lobe	19 (18.4%)	10 (27.8%)	9 (13.4%)	0.073	0.292
Hippocampus	9 (8.7%)	7 (19.4%)	2 (3.0%)	0.014	0.084
Cerebellum	3 (2.9%)	1 (2.8%)	2 (3.0%)	1.000	1.000
Abnormal EEG					
Diffuse or focal slowing	65 (63.1%)	22 (61.1%)	43 (64.2%)	0.758	0.974
Epileptiform discharges	13 (12.6%)	3 (8.3%)	10 (14.9%)	0.516	0.885
Extreme delta brush	7 (6.8%)	0 (0.0%)	7 (10.4%)	0.110	0.412

Data are presented as median (interquartile range), mean ± standard deviation, or *n* (%), as appropriate. All enrolled patients received cranial MRI and EEG examinations routinely. CSF pleocytosis was defined as CSF white blood cell count >5 cells/μL. IgG index >0.70 was considered elevated. A 24-h intrathecal IgG synthesis rate >3.3 mg/24 h was considered elevated.

QAlb, CSF/serum albumin quotient; CSF, cerebrospinal fluid; IgG, immunoglobulin G; IgGI, IgG index; 24hIgG, 24-h intrathecal IgG synthesis rate; MLR, monocyte-to-lymphocyte ratio; NLR, neutrophil-to-lymphocyte ratio; PLR, platelet-to-lymphocyte ratio; MRI, magnetic resonance imaging; EEG, electroencephalography.

**Table 4:** Disease severity, treatment strategies, and short-term outcomes in the elevated QAlb and Normal QAlb groups.

Variable	Total (*n* = 103)	Elevated QAlb group (*n* = 36)	Normal QAlb group (*n* = 67)	*p* value	FDR-adjusted *p* value
First-line immunotherapy	100 (97.1%)	35 (97.2%)	65 (100%)	1.000	1.000
Second-line therapy	4 (3.9%)	1 (2.8%)	3 (4.5%)	1.000	1.000
At peak					
mRS score	4.0 (3.0,5.0)	4.0 (3.0,5.0)	5.0 (3.0,5.0)	0.281	0.710
CASE score	12.4 ± 4.4	12.3 ± 4.5	12.5 ± 4.4	0.802	0.974
At discharge					
mRS score					
≤2	61 (59.2%)	20 (55.6%)	41 (61.2%)	0.579	0.914
≥3	42 (40.8%)	16 (44.4%)	26 (38.8%)		
CASE score	3.0 (2.0,6.0)	3.0 (2.0,5.8)	3.0 (2.0,6.0)	0.555	0.914

Data are presented as median (interquartile range), mean ± standard deviation, or *n* (%), as appropriate. Peak mRS and peak CASE score were used to assess acute disease severity. Discharge mRS and discharge CASE scores were used to assess short-term discharge outcomes. An mRS score of 0–2 was defined as a favorable short-term outcome, whereas an mRS score of 3–6 was defined as a poor short-term outcome.

QAlb, CSF/serum albumin quotient; mRS, modified Rankin Scale; CASE, Clinical Assessment Scale in Autoimmune Encephalitis.

Spearman rank correlation analysis was used to assess associations between QAlb/Qlim and IgG-related indices, CSF anti-NMDAR antibody titers, severity scores, discharge outcomes, and timing variables. Antibody titers were analyzed using natural-log-transformed denominator values of dilution titers. Reibergram analysis was used to visualize QIgG relative to QAlb and to identify patients above the Qlim(IgG) curve. The proportions of patients above Qlim(IgG) were compared between groups using Fisher’s exact test.

## Results

### Demographic and clinical characteristics

A total of 103 patients with anti-NMDAR encephalitis were included in this study. Among them, 36 patients (35.0%) exhibited a QAlb/Qlim ratio >1 and were classified into the Elevated QAlb group, while the remaining 67 patients (65.0%) had a QAlb/Qlim ratio ≤1 and were classified into the Normal QAlb group. The distributions of measured QAlb and QAlb/Qlim values are shown in Supplementary Figure S1.

The overall median age at onset was 26.0 years (19.0–34.0) and 40 patients (38.8%) were male (Table 1). Respectively, the median intervals from symptom onset to hospital admission, lumbar puncture, and initiation of immunotherapy were 10.0 (4.0–20.3), 15.0 (9.0–24.0), and 21.0 (12.0–29.5) days.

Compared with the Normal QAlb group, patients in the Elevated QAlb group had a higher proportion of male patients [58.3% (21/36) vs. 28.4% (19/67); FDR-adjusted *p* = 0.030]. Age at onset was numerically higher in the Elevated QAlb group [31.5 (21.3–39.3) vs. 24.0 (18.0–31.0) years, *p* = 0.020], but this difference did not remain significant after FDR correction (FDR-adjusted *p* = 0.109). There were no significant differences between groups in the prevalence of ovarian teratoma, history of VE, serum anti-NMDAR antibody positivity, time from onset to hospitalization, time from onset to lumbar puncture, time from onset to immunotherapy or ICU admission (all *p* > 0.05).

Regarding prodromal symptoms, headache was significantly more frequent in the Elevated QAlb group than in the Normal QAlb group [41.7% (15/36) vs. 16.4% (11/67); FDR-adjusted *p* = 0.037], whereas the prevalence of fever did not differ between groups (Table 2). The most common initial symptoms were psychiatric symptoms (68/103, 66.0%), seizures (26/103, 25.2%) and speech dysfunction (8/103, 7.8%). No significant differences were observed between the two groups in initial symptoms (all *p* > 0.05). Similarly, the core clinical manifestations identified no significant differences between groups for psychiatric symptoms, seizures, cognitive impairment, speech dysfunction, decreased level of consciousness, movement disorders, autonomic dysfunction and central hypoventilation (all *p* > 0.05).

### CSF, serum, and other ancillary examination findings

**Figure 2:**
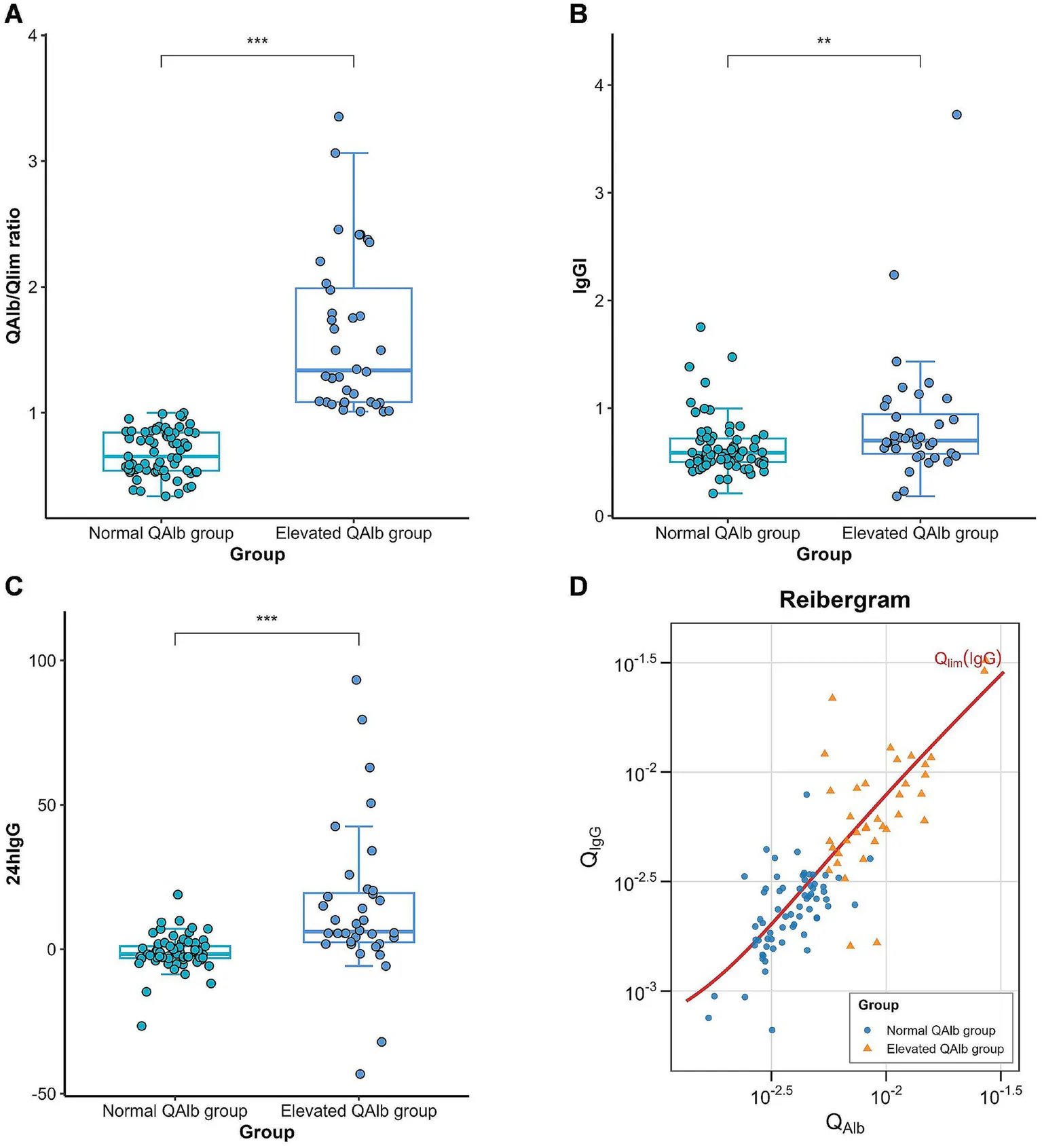
Comparison of *QAlb*/*Qlim* ratio, *IgGI*, and 24-h intrathecal IgG synthesis rate between the Elevated *QAlb* group and the Normal *QAlb* group **(A–C)** and Reibergram analysis **(D)**. **(A)** *QAlb*/*Qlim* ratio. **(B)** IgG index. **(C)** 24-h intrathecal IgG synthesis rate. **(D)** Reibergram showing the relationship between *QAlb* and *QIgG* in the two groups. The red curve represents *Qlim*(IgG), the upper reference limit for *QIgG* expected from passive blood-derived IgG transfer at a given *QAlb* level. Values above this curve indicate intrathecal IgG synthesis beyond that expected from passive transfer. In total, 13 of 103 patients (12.6%) had *QIgG* values above the *Qlim*(IgG) curve, including 9 of 36 patients (25.0%) in the Elevated *QAlb* group and 4 of 67 patients (6.0%) in the Normal *QAlb* group. *QAlb*, CSF/serum albumin quotient; *Qlim*, age-adjusted upper reference limit for *QAlb*; *QIgG*, CSF/serum IgG quotient; *IgGI*, IgG index. **p < 0.01, ***p < 0.001.

As shown in Table 3 and Figure 2, CSF protein, CSF albumin, and CSF IgG levels were significantly higher in the Elevated QAlb group than in the Normal QAlb group. Specifically, the Elevated QAlb group showed higher CSF protein [0.67 (0.53–0.96) vs. 0.37 (0.30–0.44) g/L; FDR-adjusted *p* < 0.001], CSF albumin [337.0 (256.0–504.8) vs. 159.0 (129.0–193.0) mg/L; FDR-adjusted *p* < 0.001], and CSF IgG [64.5 (47.3–98.2) vs. 25.1 (20.3–32.1) mg/L; FDR-adjusted *p* < 0.001].

Quantitative indices of intrathecal IgG synthesis were also higher in the Elevated QAlb group. Both IgGI [0.70 (0.57–1.00) vs. 0.59 (0.50–0.73); FDR-adjusted *p* = 0.037] and 24-h intrathecal IgG synthesis rate [6.09 (2.04–20.00) vs. −1.63 (−3.20 to 1.07) mg/24 h; FDR-adjusted *p* < 0.001] were significantly higher in the Elevated QAlb group. In addition, the proportion of patients with elevated 24-h intrathecal IgG synthesis rate was higher in the Elevated QAlb group [72.2% (26/36) vs. 14.9% (10/67); FDR-adjusted *p* < 0.001]. The proportion of patients with elevated IgGI showed nominal significance but did not remain significant after FDR correction [50.0% vs. 28.4%; FDR-adjusted *p* = 0.145]. In contrast, there were no significant differences in CSF pleocytosis and CSF anti-NMDAR antibody titer. Serum albumin was nominally lower in the Elevated QAlb group, but this difference did not survive FDR correction [39.98 ± 4.33 vs. 41.68 ± 3.99 g/L; FDR-adjusted *p* = 0.206]. Serum IgG levels did not differ between groups.

For peripheral blood parameters, platelet count and PLR were nominally lower in the Elevated QAlb group, but neither difference remained significant after FDR correction. Other peripheral blood parameters such as WBC count, neutrophil count, lymphocyte count, monocyte count, NLR, MLR and serum electrolytes showed no statistically significant differences.

Additionally, 32 (31.1%) of 103 patients had abnormal MRI findings and 73 (70.9%) exhibited abnormal EEG results. Hippocampal MRI abnormalities were numerically more frequent in the Elevated QAlb group than in the Normal QAlb group [19.4% (7/36) vs. 3.0% (2/67); *p* = 0.014], but this difference did not remain significant after FDR correction (FDR-adjusted *p* = 0.084). Temporal lobe abnormalities were also numerically more frequent in the Elevated QAlb group, but the difference was not statistically significant. No significant intergroup differences were observed in EEG findings, including diffuse or focal slow waves, epileptiform discharges, or extreme delta brush patterns.

### Reibergram and correlation analyses

Patients in the Elevated QAlb group tended to distribute toward higher QAlb and QIgG values compared with those in the Normal QAlb group (Figure 2D). In the Reibergram analysis, QIgG values above the Qlim(IgG) curve were observed in 13 of 103 patients (12.6%) overall. The proportion of patients above the Qlim(IgG) curve was higher in the Elevated QAlb group than in the Normal QAlb group [9/36 (25.0%) vs. 4/67 (6.0%); *p* = 0.010]. Spearman correlation analysis was performed using the QAlb/Qlim ratio. QAlb/Qlim ratio showed a positive correlation with 24-h intrathecal IgG synthesis rate (r = 0.48, *p* < 0.001) (Supplementary Figure S2).

### Disease severity, treatment, and short-term outcome

Disease severity, treatment strategies, and short-term outcomes were summarized in Table 4. Overall, 100 patients (97.1%) received first-line immunotherapy. The proportions of patients receiving first-line and second-line immunotherapy were compared between groups.

Peak mRS and peak CASE scores during hospitalization did not differ significantly between the Elevated QAlb and Normal QAlb groups. At discharge, neither the proportion of patients with a poor outcome defined by mRS ≥ 3 nor the discharge CASE score differed significantly between groups.

### Factors associated with elevated QAlb

In univariable logistic regression analyses, male sex, older age at onset, prodromal headache, and hippocampal MRI abnormalities showed nominal associations with elevated QAlb (Table 5). Time-related variables, including time from symptom onset to hospital admission, time from symptom onset to lumbar puncture and time from symptom onset to initiation of immunotherapy, as well as peak mRS, were not significantly associated with elevated QAlb (Supplementary Table S1).

**Table 5:** Logistic regression analysis of factors associated with elevated QAlb.

Variable	Univariate OR (95% CI)	*p* value	Multivariate OR (95% CI)	*p* value
Sex (Male)	3.537 (1.531–8.429)	0.004	3.029 (1.166–7.869)	0.023
Age at onset	1.033 (1.002–1.066)	0.043	1.024 (0.988–1.062)	0.199
Prodromal headache	3.636 (1.456–9.391)	0.006	4.089 (1.480–11.292)	0.007
Hippocampal MRI abnormality	7.845 (1.770–54.863)	0.013	6.745 (1.174–38.756)	0.032

Variables with *p* < 0.05 in univariable logistic regression analyses were entered into the exploratory multivariable logistic regression model. The dependent variable was elevated QAlb. QAlb, CSF/serum albumin quotient; OR, odds ratio; CI, confidence interval; MRI, magnetic resonance imaging.

Variables with *p* < 0.05 in univariable analysis were entered into the multivariate logistic regression model. After adjustment, male sex (OR = 3.029, 95% CI: 1.166–7.869; *p* = 0.023), prodromal headache (OR = 4.089, 95% CI: 1.480–11.292; *p* = 0.007), and hippocampal MRI abnormality (OR = 6.745, 95% CI: 1.174–38.756; *p* = 0.032) remained associated with elevated QAlb (Table 5).

Age at onset was no longer significantly associated with elevated QAlb after multivariable adjustment (*p* = 0.199). In sensitivity analyses, male sex and prodromal headache remained consistently associated with elevated QAlb after separately adding peak mRS, peak CASE score, and individual timing variables to the main model (Supplementary Table S2). Hippocampal MRI abnormality remained directionally associated with elevated QAlb, but the association became borderline after adjustment for time from symptom onset to initiation of immunotherapy (OR = 5.719, 95% CI: 1.001–32.674; *p* = 0.050). Given the small number of hippocampal MRI abnormalities and the lack of significance after FDR correction in both between-group comparisons and the full univariable logistic regression analysis, this imaging finding should be interpreted as exploratory.

## Discussion

In this retrospective cohort of patients with anti-NMDAR encephalitis, we investigated the clinical significance of elevated age-adjusted QAlb by stratifying patients into an Elevated QAlb group and a Normal QAlb group. Our findings demonstrated that elevated QAlb was observed in approximately one-third of the cohort, consistent with previously reported prevalence rates (Yu et al., 2021; Lai et al., 2022). Elevated QAlb was associated with higher quantitative indices of intrathecal IgG synthesis and was more frequently observed in male patients and those with prodromal headache. Hippocampal MRI abnormalities were also more common among patients with elevated QAlb, although this association was based on a small number of events and was attenuated in sensitivity analysis. In contrast, elevated QAlb was not associated with CSF anti-NMDAR antibody titers, acute disease severity, ICU admission, or discharge outcome. Collectively, these findings suggest that age-adjusted QAlb elevation may help characterize a CSF biochemical pattern associated with increased quantitative indices of total intrathecal IgG synthesis, rather than serving as a standalone indicator of acute disease burden or short-term discharge outcome.

Male sex remained associated with elevated QAlb after multivariable adjustment and across sensitivity analyses. Sex-related differences in QAlb have been reported in healthy individuals and in patients with neurological and psychiatric disorders (Castellazzi et al., 2020; Meixensberger et al., 2020; Castellazzi et al., 2022), suggesting that this association is not specific to anti-NMDAR encephalitis. Biological factors related to sex hormones, vascular physiology, CSF turnover, and blood-CSF interface function may contribute to these differences. Experimental studies have suggested that estrogen-related signaling may influence endothelial tight-junction organization and inflammatory barrier permeability (Maggioli et al., 2016; Greene et al., 2019). Accordingly, the association between male sex and elevated QAlb observed in our cohort may be partially attributable to inherent physiological baseline differences between sexes rather than representing evidence of a disease-specific pathogenic mechanism. Further studies are needed to clarify how these baseline sex-related differences interact with inflammatory and CSF-related changes during autoimmune encephalitis. Age at onset was nominally associated with elevated QAlb in univariable analysis but lost significance in the multivariable model. This attenuation may partly reflect the fact that elevated QAlb was defined using the age-adjusted QAlb/Qlim ratio, in which the expected physiological increase in QAlb with age is already incorporated. Therefore, chronological age may not exert an additional independent effect after age adjustment and inclusion of sex and other clinical variables.

Prodromal headache was more frequently observed in the Elevated QAlb group and remained associated with elevated QAlb in multivariable and sensitivity analyses. Previous evidence suggests that headache is an epiphenomenon of NMDAR autoimmunity, representing a non-infectious intrathecal inflammatory response (Tominaga et al., 2018), which may reflect early disturbance of the CSF inflammatory microenvironment or imbalance between BCB function and CSF homeostasis. However, headache is a highly nonspecific symptom and can be observed in infectious, inflammatory, vascular, and functional neurological disorders. In addition, detailed headache characteristics, including location, duration, intensity, associated symptoms, and temporal relationship to encephalitic manifestations, and response to analgesics, were not systematically or consistently documented in the retrospective records. Therefore, prodromal headache should be interpreted as a nonspecific clinical correlate of elevated QAlb rather than as a diagnostic marker or mechanistic biomarker of altered blood-to-CSF albumin transfer.

One of the principal findings of this study was the association between elevated QAlb and higher quantitative indices of intrathecal IgG synthesis. Patients with elevated QAlb had higher CSF IgG concentrations, IgGI values, and calculated 24-h intrathecal IgG synthesis rates, as well as a higher proportion of elevated 24-h intrathecal IgG synthesis rate. Reibergram analysis further showed that a higher proportion of patients in the Elevated QAlb group had QIgG values above the Qlim(IgG) curve, supporting increased total intrathecal IgG synthesis beyond passive blood-derived transfer in a subset of patients. These findings are consistent with previous reports showing that elevated QAlb may coexist with increased quantitative indices of intrathecal IgG synthesis in anti-NMDAR encephalitis and other neuronal surface antibody-associated encephalitides (Yu et al., 2021; Lai et al., 2022). However, the interpretation of these associations requires caution. Calculated IgG synthesis indices are not completely independent of QAlb. IgGI is derived from QIgG relative to QAlb, and the 24-h intrathecal IgG synthesis rate also incorporates CSF and serum albumin measurements. Similarly, Reibergram interpretation evaluates QIgG against QAlb-dependent reference limits. Therefore, the observed correlations between QAlb/Qlim and calculated IgG synthesis indices may be partly influenced by mathematical coupling and should not be interpreted as proof of a direct causal relationship between QAlb elevation and intrathecal IgG production. Nevertheless, QAlb and IgG synthesis indices represent different clinical dimensions. QAlb primarily reflects blood-to-CSF albumin transfer and BCB function, whereas IgGI, 24-h intrathecal IgG synthesis rate, and Reibergram-based interpretation aim to assess whether CSF IgG is increased beyond what would be expected from passive blood-derived transfer. Thus, the coexistence of elevated QAlb and increased IgG synthesis indices suggests a CSF biochemical pattern involving both altered blood-to-CSF albumin transfer and increased total intrathecal IgG synthesis. Previous studies have implicated B-cell-related chemokines and cytokines, such as CXCL13, BAFF, APRIL, and IL-6, in intrathecal humoral immune activation in anti-NMDAR encephalitis (Leypoldt et al., 2015; Deng et al., 2017; Byun et al., 2016). However, these mediators were not measured in the present cohort, and their role in the observed CSF biochemical pattern remains hypothetical.

Notably, CSF anti-NMDAR antibody titers were not significantly different between the Elevated QAlb and Normal QAlb groups. This apparent dissociation may reflect the distinction between total intrathecal IgG synthesis and antigen-specific antibody production. The IgGI, 24-h IgG synthesis rate, and Reibergram-based interpretation assess total intrathecal IgG synthesis or CSF IgG enrichment beyond passive blood-derived transfer, whereas routine CSF anti-NMDAR antibody titers are semi-quantitative antigen-specific measurements. The latter may also be influenced by antibody affinity, epitope specificity, functional activity, assay sensitivity, dilution intervals, and the number and activity of local anti-NMDAR antibody-secreting cells. Therefore, enhanced quantitative indices of total intrathecal IgG synthesis do not necessarily correspond to higher measured CSF anti-NMDAR antibody titers.

Hippocampal MRI abnormalities were more frequent in patients with elevated QAlb. The hippocampus contains abundant NMDARs and depends on NMDAR-mediated synaptic transmission and plasticity (Dalmau et al., 2007; Hughes et al., 2010). The hippocampus, particularly the CA1 subfield, is selectively vulnerable to ischemic–hypoxic injury, inflammation, and the excessive metabolic demands associated with prolonged seizure activity (Bartsch et al., 2015; Bartsch and Wulff, 2015). These features provide biological plausibility for hippocampal involvement in anti-NMDAR encephalitis. However, only nine patients in the overall cohort had hippocampal MRI abnormalities, and the corresponding confidence interval in the multivariable model was wide. Moreover, the association was attenuated to borderline significance after adjustment for the interval from symptom onset to immunotherapy. MRI abnormalities may also be influenced by seizure burden, disease stage, local inflammatory activity, imaging protocol, and the timing of MRI relative to lumbar puncture and treatment. Accordingly, the relationship between elevated QAlb and hippocampal MRI abnormalities should be considered an exploratory imaging association rather than evidence that altered BCB function directly causes hippocampal injury.

Platelet count and PLR were nominally lower in the Elevated QAlb group, but these findings did not remain significant after FDR correction and should therefore be regarded as exploratory. Platelet-related indices may be influenced by systemic inflammation, infection, stress response, medication exposure, comorbidities, or other nonspecific clinical factors. In addition, PLR is a composite peripheral inflammatory marker and may be difficult to interpret when driven primarily by variation in platelet count. Therefore, these hematological findings should not be interpreted as evidence of a QAlb-specific mechanism and require validation in larger independent cohorts.

In the study, no significant intergroup differences were observed in acute severity measures or short-term functional outcomes at discharge. Previous studies have reported inconsistent findings regarding the prognostic significance of QAlb in anti-NMDAR encephalitis. Yu et al. (2021) found that QAlb-defined barrier dysfunction was associated with decreased consciousness, ICU admission, higher inflammatory indices, enhanced intrathecal IgG synthesis, and worse 2-month outcome in anti-NMDAR encephalitis. In contrast, Lai et al. (2022) reported no significant difference in discharge or last-follow-up mRS within the anti-NMDAR encephalitis subgroup, despite an association between elevated QAlb and poor prognosis in the overall mixed-antibody cohort. These discrepancies may be explained by differences in cohort composition, disease severity distribution, QAlb definition, sampling timing, treatment timing, and outcome assessment. Our use of age-adjusted QAlb elevation defined by the QAlb/Qlim ratio may identify a different subgroup from studies using unadjusted or fixed-threshold QAlb definitions. Moreover, discharge outcomes may be influenced by treatment-related factors, including time to immunotherapy, escalation of immunotherapy, ICU care, and supportive management; residual confounding by indication cannot be excluded in this retrospective cohort. Therefore, the lack of association between elevated QAlb and discharge outcomes should be interpreted cautiously. Discharge mRS and CASE scores reflect short-term status only and may not capture prolonged recovery, cognitive or psychiatric sequelae, relapse risk, or delayed treatment response. Thus, our findings suggest that age-adjusted QAlb elevation was not associated with acute disease severity or short-term discharge outcomes in this cohort, but they do not exclude a potential relationship between QAlb/Qlim and long-term prognosis.

This study has several limitations. First, this was a single-center retrospective study conducted in a tertiary hospital, which may introduce selection and referral bias and limit the generalizability of the findings. Second, QAlb is an indirect functional marker of albumin transfer and barrier/CSF-related dynamics. Direct measures of barrier integrity, such as dynamic contrast-enhanced MRI, endothelial injury markers, tight-junction-related biomarkers or choroid plexus-related assessments were not available. Third, although paired CSF and serum samples were collected before immunotherapy, serial CSF sampling was not performed; therefore, dynamic changes in QAlb and intrathecal IgG synthesis during the disease course could not be evaluated. Fourth, EEG findings should be interpreted with caution because we were unable to systematically control for the timing of recordings relative to symptom onset, ongoing clinical seizures, sedative medication use, or ICU admission. Finally, the multivariable logistic regression model should be interpreted cautiously because only 36 patients had elevated QAlb. Despite restricting the number of covariates, overfitting cannot be excluded, particularly for estimates with wide confidence intervals, such as hippocampal MRI abnormalities.

## Conclusion

In conclusion, age-adjusted QAlb elevation, defined by the QAlb/Qlim ratio, was associated with increased quantitative indices of total intrathecal IgG synthesis in patients with anti-NMDAR encephalitis, but not with CSF anti-NMDAR antibody titers, acute disease severity, or short-term discharge outcomes. Exploratory associations with male sex, prodromal headache, and hippocampal MRI abnormalities were observed, but these findings require cautious interpretation and independent validation. Overall, QAlb/Qlim may provide an accessible measure for characterizing CSF biochemical heterogeneity related to blood-to-CSF albumin transfer and total intrathecal IgG synthesis. However, the present findings do not support stronger mechanistic or prognostic conclusions. Prospective multicenter studies with serial paired serum–CSF sampling, standardized neuroimaging, direct immune and barrier-related biomarkers, and long-term clinical follow-up are needed to determine the biological and long-term prognostic significance of QAlb/Qlim in anti-NMDAR encephalitis.

## Data Availability

The raw data supporting the conclusions of this article will be made available by the authors, without undue reservation.
