# Supplementary material for: Clinical and immunological correlates of elevated age-adjusted QAlb in anti-NMDAR encephalitis
Source: Front Hum Neurosci. 2026 Aug 7;20:1914297. doi: 10.3389/fnhum.2026.1914297 (PMC13493388; doi:10.3389/fnhum.2026.1914297)
Supplement: Supplementary file 1 [file Data_Sheet_1.docx]

**Supplementary Figure 1 Distribution of QAlb and QAlb/Qlim ratio in patients with anti-NMDAR encephalitis.**

A, Histogram showing the distribution of QAlb values in the entire cohort. QAlb values are displayed as QAlb ×10⁻³. B, Histogram showing the distribution of QAlb/Qlim ratio. The dashed vertical line indicates QAlb/Qlim = 1, corresponding to the age-adjusted upper reference limit used to define elevated QAlb.

**Supplementary Table 1 Full univariable logistic regression analysis of factors associated with elevated QAlb**

| Variable | β | SE | p value | FDR-adjusted p value | OR (95%CI) |
| --- | --- | --- | --- | --- | --- |
| Sex (Male) | 1.263 | 0.433 | 0.004 | 0.129 | 3.537 (1.531-8.429) |
| Age at onset,years | 0.032 | 0.016 | 0.043 | 0.430 | 1.033 (1.002-1.066) |
| Ovarian teratoma | -1.206 | 0.799 | 0.131 | 0.512 | 0.299 (0.063-1.433) |
| History of VE | 0.069 | 0.664 | 0.917 | 0.970 | 1.071 (0.292-3.936) |
| Positive for serum antibody | 0.171 | 0.456 | 0.707 | 0.959 | 1.187 (0.486-2.901) |
| Time from onset to hospitalization, days | 0.011 | 0.013 | 0.392 | 0.790 | 1.011 (0.985-1.038) |
| Time from onset to  lumbar puncture, days | 0.020 | 0.013 | 0.118 | 0.512 | 1.021 (0.995-1.047) |
| Time from onset to immunotherapy, days | 0.023 | 0.013 | 0.086 | 0.462 | 1.023 (0.997-1.051) |
| ICU admission | -0.706 | 0.459 | 0.124 | 0.512 | 0.494 (0.193-1.184) |
| Prodromal headache | 1.291 | 0.472 | 0.006 | 0.129 | 3.636 (1.456-9.391) |
| Prodromal fever | 0.308 | 0.448 | 0.492 | 0.837 | 1.361 (0.557-3.268) |
| Initial symptom |  |  |  |  |  |
| Psychiatric symptom | 0.045 | 0.437 | 0.919 | 0.970 | 1.046 (0.447-2.509) |
| Seizures | -0.020 | 0.476 | 0.967 | 0.970 | 0.980 (0.373-2.458) |
| Speech dysfunction | 0.120 | 0.761 | 0.875 | 0.970 | 1.127 (0.220-4.887) |
| Clinical presentation |  |  |  |  |  |
| Psychiatric symptom | -0.120 | 0.761 | 0.875 | 0.970 | 0.887 (0.205-4.540) |
| Seizures | -0.356 | 0.440 | 0.419 | 0.790 | 0.700 (0.296-1.678) |
| Cognitive impairment | -0.550 | 0.459 | 0.231 | 0.621 | 0.577 (0.234-1.433) |
| Speech dysfunction | -0.201 | 0.414 | 0.628 | 0.931 | 0.818 (0.361-1.842) |
| Movement disorder | -0.331 | 0.415 | 0.425 | 0.790 | 0.718 (0.316-1.622) |
| Autonomic dysfunction | -0.590 | 0.433 | 0.173 | 0.572 | 0.554 (0.236-1.301) |
| Decreased level of consciousness | -0.624 | 0.447 | 0.163 | 0.572 | 0.536 (0.216-1.262) |
| Central hypoventilation | -0.185 | 0.544 | 0.733 | 0.959 | 0.831 (0.269-2.337) |
| CSF antibody titer | -0.059 | 0.348 | 0.865 | 0.970 | 0.942 (0.476-1.865) |
| Hematological parameters |  |  |  |  |  |
| WBC | -0.032 | 0.064 | 0.620 | 0.931 | 0.969 (0.850-1.097) |
| Neutrophil | -0.036 | 0.066 | 0.591 | 0.931 | 0.965 (0.843-1.096) |
| Monocyte | -0.028 | 0.753 | 0.970 | 0.970 | 0.972 (0.214-4.225) |
| Lymphocyte | 0.095 | 0.302 | 0.752 | 0.959 | 1.100 (0.602-1.994) |
| Platelet | -0.006 | 0.003 | 0.057 | 0.430 | 0.994 (0.987-1.000) |
| MLR | -0.592 | 0.769 | 0.441 | 0.790 | 0.553 (0.100-2.212) |
| NLR | -0.060 | 0.046 | 0.192 | 0.590 | 0.942 (0.848-1.020) |
| PLR | -0.006 | 0.003 | 0.060 | 0.430 | 0.994 (0.987-1.000) |
| Na^+^, mmol/L | -0.036 | 0.043 | 0.394 | 0.790 | 0.964 (0.887-1.049) |
| K^+^, mmol/L | -0.258 | 0.603 | 0.669 | 0.959 | 0.773 (0.237-2.521) |
| Ca^2+^, mmol/L | -1.564 | 1.760 | 0.374 | 0.790 | 0.209 (0.007-6.591) |
| Abnormal MRI |  |  |  |  |  |
| Frontal lobe | 0.389 | 0.585 | 0.506 | 0.837 | 1.475 (0.449-4.632) |
| Parietal lobe | 0.981 | 0.794 | 0.217 | 0.621 | 2.667 (0.556-14.223) |
| Temporal lobe | 0.908 | 0.517 | 0.079 | 0.462 | 2.479 (0.898-6.957) |
| Hippocampus | 2.060 | 0.832 | 0.013 | 0.186 | 7.845 (1.770-54.863) |
| Cerebellum | -0.074 | 1.243 | 0.952 | 0.970 | 0.929 (0.042-10.024) |
| Abnormal EEG |  |  |  |  |  |
| Diffuse or focal slowing | -0.131 | 0.426 | 0.758 | 0.959 | 0.877 (0.381-2.045) |
| Epileptiform discharges | -0.657 | 0.694 | 0.343 | 0.790 | 0.518 (0.110-1.836) |
| Peak mRS | -0.194 | 0.212 | 0.360 | 0.790 | 0.823 (0.543-1.249) |
| Peak CASE | -0.012 | 0.047 | 0.800 | 0.970 | 0.988 (0.901-1.084) |

The dependent variable was elevated QAlb. FDR-adjusted p values were calculated using the Benjamini–Hochberg method.

QAlb, CSF/serum albumin quotient; OR, odds ratio; CI, confidence interval; MLR, monocyte-to-lymphocyte ratio; NLR, neutrophil-to-lymphocyte ratio; PLR, platelet-to-lymphocyte ratio; MRI, magnetic resonance imaging; EEG, electroencephalography; mRS, modified Rankin Scale; CASE, Clinical Assessment Scale in Autoimmune Encephalitis.

**Supplementary Table 2 Sensitivity analyses of multivariable logistic regression models for elevated QAlb**

| Model | Additional adjustment† | Male sex OR (95% CI) | p value | Prodromal headache OR (95% CI) | p value | Hippocampal MRI abnormality OR (95% CI) | p value |
| --- | --- | --- | --- | --- | --- | --- | --- |
| Main model* | None | 3.029 (1.166-7.869) | 0.023 | 4.089 (1.480-11.292) | 0.007 | 6.745 (1.174-38.756) | 0.032 |
| Model 1 | Peak mRS | 2.917 (1.090-7.802) | 0.033 | 4.635 (1.623-13.232) | 0.004 | 8.008 (1.309-49.008) | 0.024 |
| Model 2 | Peak CASE | 3.123 (1.190-8.195) | 0.021 | 4.113 (1.480-11.431) | 0.007 | 7.919 (1.281-48.960) | 0.026 |
| Model 3 | Time from onset to hospitalization | 2.964 (1.139-7.718) | 0.026 | 4.597 (1.558-13.565) | 0.006 | 6.000 (1.023-35.187) | 0.047 |
| Model 4 | Time from onset to lumbar puncture | 3.146 (1.167-8.482) | 0.024 | 5.089 (1.698-15.254) | 0.004 | 6.179 (1.063-35.905) | 0.043 |
| Model 5 | Time from onset to immunotherapy | 3.016 (1.112-8.178) | 0.030 | 3.940 (1.353-11.479) | 0.012 | 5.719 (1.001-32.674) | 0.050 |

*The main model included age at onset, male sex, prodromal headache and hippocampal MRI abnormality.

†Each sensitivity model was additionally adjusted for one disease severity or timing-related variable. Additional variables were added separately to avoid overfitting and potential collinearity among time-related variables.

QAlb, CSF/serum albumin quotient; OR, odds ratio; CI, confidence interval;MRI, magnetic resonance imaging; mRS, modified Rankin Scale; CASE, Clinical Assessment Scale in Autoimmune Encephalitis.

**Supplementary Figure 2 Spearman correlation matrix of QAlb/Qlim ratio and clinical-immunological variables in anti-NMDAR encephalitis.**
Spearman correlation analysis was performed to evaluate the relationships among QAlb/Qlim ratio, intrathecal IgG synthesis indices, CSF anti-NMDAR antibody titer, disease severity scores, short-term outcome measures, and timing-related variables. Numbers in each cell indicate Spearman’s correlation coefficients. Red indicates positive correlations, whereas blue indicates negative correlations. Anti-NMDAR antibody titers were converted to ordinal numeric values before analysis.

QAlb, CSF/serum albumin quotient; Qlim, age-corrected upper reference limit; CASE, Clinical Assessment Scale in Autoimmune Encephalitis; mRS, modified Rankin Scale. *P <0.05, **P <0.01, ***P <0.001.
